# *Yersinia pestis*-Induced Mitophagy That Balances Mitochondrial Homeostasis and mROS-Mediated Bactericidal Activity

**DOI:** 10.1128/spectrum.00718-22

**Published:** 2022-06-06

**Authors:** Yang Jiao, Shiyang Cao, Yuan Zhang, Yafang Tan, Yazhou Zhou, Tong Wang, Yang You, Hongyan Chen, Yifan Ren, Ruifu Yang, Zongmin Du

**Affiliations:** a State Key Laboratory of Pathogen and Biosecurity, Beijing Institute of Microbiology and Epidemiology, Beijing, China; Emory University School of Medicine

**Keywords:** mitochondrial dysfunction, mROS, mitophagy, *Yersinia pestis*, YopH, plague

## Abstract

Manipulating mitochondrial homeostasis is essential for host defense against infection and pathogen survival in cells. This study reports for the first time that Y. pestis infection caused mitochondria damage that subsequently leads to the activation of Pink1/Parkin-independent mitophagy in macrophage, and the effector YopH from the type III secretion system was required for these effects. The generation of mitochondrial reactive oxygen species (mROS) by damaged mitochondria enhances the antibacterial activity of macrophages against Y. pestis and promotes apoptosis of the infected cells. Therefore, Y. pestis-induced mitophagy was employed to eliminate dysfunctional mitochondria and relieve the mROS accumulation. This study reveals a novel role for YopH of Y. pestis in damaging host macrophage mitochondria during plague infection and underlines the vital role of mitophagy in maintaining mitochondrial homeostasis by clearing bacteria-damaged mitochondria. The results show that mitophagy or mitochondrial fission manipulation could be used as a new strategy to treat plague.

**IMPORTANCE** Y. pestis, the pathogen of plague, also known as the “Black Death,” has caused millions of deaths throughout history. This study reports that Y. pestis infection induces mitochondrial fragmentation and abnormal mROS accumulation, and releases mitochondrial contents into the cytoplasm in macrophages. mROS promotes the antibacterial activity of macrophages against Y. pestis and increases apoptosis of the infected cells. PINK-Parkin-independent mitophagy is activated to balance mitochondrial homeostasis and mROS-induced bactericidal activity in Y. pestis*-*infected macrophages. These findings deepen the understanding of Y. pestis pathogenesis on mitochondria damage to disturb the host cellular immune elimination. Manipulating mitophagic activity or mitochondrial fission may be a novel therapeutic approach to treat plague.

## INTRODUCTION

Mitochondria are essential organelles involved in multiple functions, from energy production and fatty acid oxidation to regulation of calcium (Ca^2+^) homeostasis and programmed cell death. Moreover, accumulating evidence demonstrates the crucial roles of mitochondrial reactive oxygen species (mROS) in phagocytes in killing pathogens. The major bactericidal effects of mROS are oxidative burst induction and hypochlorous acid generation (1–4). On the other hand, pathogens employ diverse strategies to disrupt mitochondrial homeostasis, including damage to mitochondrial dynamics, modulation of mitochondrial metabolism, subversion of mitochondrial quality control, and so on, resulting in cell death and tissue damage and promotion of pathogen survival and proliferation (5–9). The selective degradation of mitochondria by autophagy, termed mitophagy, is an evolutionarily conserved mechanism for maintaining mitochondrial dynamics and homeostasis. In mitophagy, infection-induced dysfunctional mitochondria are degraded to prevent the accumulation of potentially harmful mitochondrial content that could cause excessive inflammatory responses (10–12). Strikingly, some pathogens benefit by hijacking mitophagy to influence the host cells’ metabolism and physiology, facilitating their survival (8, 13). For instance, listeriolysin O (LLO) in Listeria monocytogenes induces oligomerization of NLRX1 (NOD-like receptor X1, localized to the outer mitochondrial membrane) to promote binding of its LIR (LC3-interacting region) motif to LC3 for induction of mitophagy. Subsequently, induced mitophagy decreases mROS production to promote *Listeria* replication (6).

Yersinia pestis is the causative agent of plague and has caused millions of deaths throughout history (14, 15). This lethal pathogen possesses a range of *Yersinia* outer proteins (Yops) that can be delivered into the host cytosol via a type III secretion system (T3SS). Y. pestis utilizes these major virulence factors to subvert host defenses and facilitate survival (16, 17). One of these Yops, YopH, is a potent tyrosine phosphatase which dephosphorylates various functionally distinct substrates. Injection of YopH into phagocytic cells leads to the loss of focal adhesions and the inhibition of integrin-mediated bacterial phagocytosis (18–20). YopH also suppresses T and B lymphocyte activation to block the adaptive immune response (21). What draws more of our attention is the fact that YopH induces apoptosis by affecting the mitochondrial membrane potential (ΔΨm) (22). Decreased ΔΨm is a typical feature of mitochondrial dysfunction. Nevertheless, the impacts of Y. pestis infection on mitochondria are far from clear, and no study has reported induction of mitophagy in a plague infection.

This study reports that plague infection induces mitochondrial damage in host immune cells by the YopH protein, followed by mitophagy activation. This finding indicates the vital role of mitophagy in maintaining mitochondrial homeostasis as a strategy to suppress bacteria-induced mitochondria dysfunction, and suggests manipulation of mitophagy or mitochondrial fission as a new strategy to treat infectious diseases.

## RESULTS

### *Y. pestis* causes severe mitochondrial damage in a T3SS-dependent manner.

**FIG 1:**
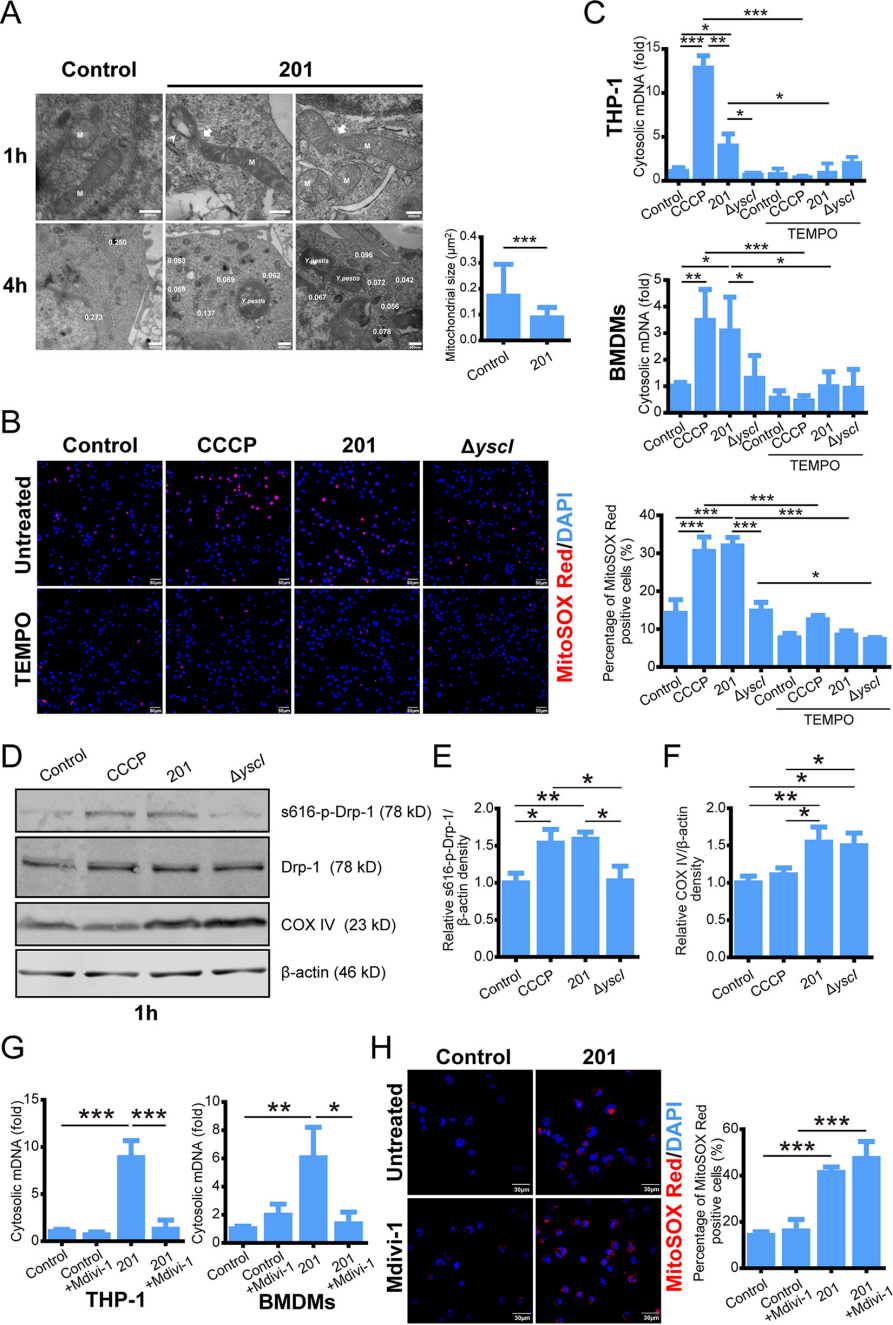
Yersinia pestis infection causes severe mitochondrial damage, including mitochondrial fission, mitochondrial reactive oxygen species (mROS) accumulation, and mitochondrial DNA (mDNA) release. (A) Electron micrographs of THP-1 cells infected with strain 201 (multiplicity of infection [MOI] = 40 for 1 or 4 h). Arrowhead indicates vacuolar degeneration in mitochondria; arrow indicates ongoing mitochondrial fission. M indicates mitochondria. Scale bar = 1 h, 200 nm; 4 h, 400 nm. White dots indicate that mitochondrial sizes (μm^2^) were determined. Data are reported as the mean ± standard deviation (SD) from five fields. A two-tailed unpaired Student’s *t* test was used to measure significance. ***, adjusted *P < *0.001. (B) THP-1 cells, untreated or pretreated with Mito-TEMPO (500 μM) for 1 h, were infected with strain 201 and Δ*yscI* (MOI 40 for 4 h) and stained with MitoSOX Red and 4’,6-diamidino-2-phenylindole (DAPI). Cyanide 3-chlorophenylhydrazone (CCCP) treatment (30 μM, 4 h) was used as a positive control. Representative confocal images from three independent experiments are shown. Scale bar = 50 μm. The relative % of the MitoSOX Red positive cells were determined (*n* ≈ 200). Data are reported as the mean ± SD from three independent experiments. One-way analysis of variance (ANOVA) followed by Tukey’s multiple-comparison test was used to measure significance. *, adjusted *P < *0.05 and ***, adjusted *P < *0.001. (C) THP-1 cells/mouse bone marrow-derived macrophages, untreated or pretreated with Mito-TEMPO (500 μM) for 1 h, were infected with strain 201 and Δ*yscI* (MOI = 40 for 4 h). CCCP (30 μM, 4 h) treatment was used as a positive control. Cytosolic mDNA relative to nuclear DNA was analyzed by quantitative PCR (qPCR). Data are shown as the mean ± SD from three independent experiments. One-way ANOVA followed by Tukey’s multiple-comparison test was used to measure significance. *, adjusted *P* < 0.05; **, adjusted *P < *0.01; and ***, adjusted *P < *0.001. (D) THP-1 cells were infected with the indicated strains (MOI = 40 for 1 h) or stimulated with CCCP (30 μM) for 1 h. Cell lysates were analyzed for S616-p-Drp1, Drp-1, or COX IV by immunoblotting. Relative densities of S616-p-Drp1 (E) and COX IV (F) were determined using Quantity One 4.6.2 software (Bio-Rad, Hercules, CA). *n* = 3. One-way ANOVA followed by Tukey’s multiple-comparison test was used to measure significance. *, adjusted *P < *0.05 and **, adjusted *P < *0.01. (G) THP-1 cells/mouse BMDMs, untreated or pretreated with Mdivi-1 (50 μM) for 1 h, were infected with strain 201 (MOI = 40 for 4 h). The level of cytosolic mDNA relative to nuclear DNA was analyzed by qPCR. Data are shown as the mean ± SD from three independent experiments. One-way ANOVA followed by Tukey’s multiple-comparison test was used to measure significance. *, adjusted *P < *0.05; **, adjusted *P < *0.01; and ***, adjusted *P < *0.001. (H) THP-1 cells untreated or pretreated with Mdivi-1 (50 μM) for 1 h were infected with strain 201 (MOI = 40 for 4 h) and stained with MitoSOX Red. Representative confocal images from three independent experiments are shown. Scale bar = 30 μm. The relative % of MitoSOX Red-positive cells was determined (*n* ≈ 200). Data are reported as the mean ± SD from three independent experiments. One-way ANOVA followed by Tukey’s multiple-comparison test was used to measure significance. ***, adjusted *P < *0.001.

Using transmission electron microscopy, we found that fragmented mitochondria and mitochondrial vacuolar degeneration occurred in THP-1 cells infected with strain 201 (Y. pestis Microtus strain, highly virulent to mice but avirulent to humans) (23), in contrast to the uninfected cells, which displayed elongated and tubular mitochondria. The mitochondria fission appeared to begin at 1 h postinfection (hpi) or even earlier, and mitochondrial fragmentation was completed by 4 hpi since almost no elongated mitochondrial could be found at this time point (Fig. 1A). Similarly, the fragmented mitochondria also appeared in U937 cells infected with strains 201 or 141 (Y. pestis biovar Antiqua strain, highly virulent in both mice and humans) (24) (Fig. S1A in the supplemental material). The mitochondrial fragmentation post-Y. pestis infection indicated that mitochondrial dysfunction and damage occur during infection. Next, we examined the production of reactive oxygen by mitochondria after strain 201 or Δ*yscI* infection (Fig. 1B). YscI is a rod-like protein that connects the needle and the center of the basal body of the injectisome, the lack of which leads to the failed translocation of T3SS. After strain 201 infection or carbonyl cyanide 3-chlorophenylhydrazone (CCCP, a mitophagy inducer that suppresses the expression of some electron transport chain proteins) stimulation, there was a significant increase in mROS production, but not in Δ*yscI*-infected THP-1 cells. In addition, treatment with Mito-TEMPO, a specific inhibitor of mitochondrial reactive oxygen production, lowered mROS. Similarly, mROS accumulation also appeared in mouse bone marrow-derived macrophages (BMDMs) infected with strain 201 (Fig. S1B). Third, we assayed for cytoplasmic mitochondrial DNA (mDNA) release in THP-1 cells and mouse BMDMs infected with strain 201 or Δ*yscI*. We found a marked increase in the ratio of mitochondrial to nuclear cytoplasmic DNA in cells infected with strain 201 or treated with CCCP compared to that in cells infected with strain Δ*yscI*. Clearing mROS with Mito-TEMPO significantly inhibited mDNA release (Fig. 1C), indicating that cytoplasmic mDNA release might be induced by the mROS accumulation post-Y. pestis infection, as reported by Jabir et al. (25). Altogether, the functional role of T3SS is indispensable for Y. pestis to cause mitochondrial damage in macrophages, including mitochondrial fragmentation, abnormal mROS accumulation, and the release of mitochondrial contents into the cytoplasm.

Mitochondrial fission is primarily mediated by dynamin-related protein 1 (Drp-1). Phosphorylation at serine 616 induces Drp-1 localization at the fission site on the mitochondrion (26). Hence, this study analyzed Drp-1 phosphorylation post-Y. pestis infection. Levels of S616-p-Drp1 increased in the 201-infected cells in comparison to that in the Δ*yscI*- and mock-infected cells at 1 hpi (Fig. 1D and E). These results are concurrent with the electron microscopy observation that mitochondria fission occurred in 201-infected THP-1 cells at 1 hpi (Fig. 1A). Moreover, mitochondrial dysfunction can include abnormal expression of cytochrome c oxidase (COX), leading to ROS accumulation (27, 28). We found that Y. pestis infection enhanced the expression of cytochrome c oxidase IV (COX IV, the terminal enzyme of the respiratory chain in complex IV) in THP-1 cells, compared to a lack of marked effect on COX IV following CCCP treatment (Fig. 1D and F). A functional T3SS was unnecessary for Y. pestis to promote COX IV expression. To test the dependence of Drp-1 activity on Y. pestis-induced mitochondrial dysfunction, we used the Drp-1 inhibitor Mdivi-1, which attenuates mitochondrial fission. Following infection with strain 201, this Mdivi-1 treatment markedly decreased mDNA release in THP-1 cells and mouse BMDMs (Fig. 1G) but did not affect mROS accumulation (Fig. 1H). These results indicated that although Drp-1 activity is dispensable for mROS accumulation, Drp-1 is required for the release of mitochondrial contents into the cytoplasm during Y. pestis infection. Our data demonstrate that Y. pestis infection causes severe mitochondrial damage and mROS accumulation, and Drp-1 is required for the release of mitochondrial contents.

### *Y. pestis*-induced mitochondrial damage activates mitophagy.

**FIG 2:**
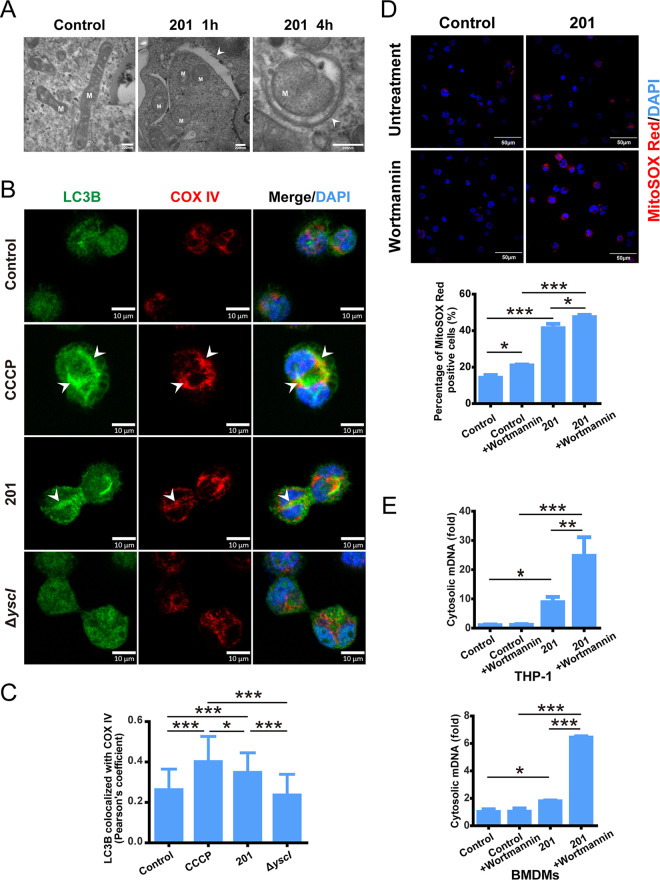
Y. pestis infection induces mitophagy in a type III secretion system (T3SS)-dependent manner. (A) Electron micrographs of double-membrane structure engulfing mitochondria (phagophore or mitophagosome) in THP-1 cells infected with strain 201 (MOI = 40 for 1 or 4 h). Arrowheads indicate phagophores or mitophagosomes, M indicates mitochondria. Scale bar = 200 nm. (B) THP-1 cells were infected with the indicated strains (MOI = 40 for 4 h) or stimulated with CCCP (30 μM) for 4 h, then stained for LC3B (green) and mitochondrial marker protein COX IV (red). Nuclei were stained with DAPI. Representative confocal images from two independent experiments were shown. Arrowheads indicated the colocalization of LC3B with mitochondria. Scale bar = 10 μm. (C) Pearson's coefficient values for colocalization of LC3B and COX IV in (B). The average Pearson's coefficients ± SD were calculated for 50 cells from two independent experiments by ImageJ 1.53e software (National Institutes of Health). One-way ANOVA followed by Tukey’s multiple-comparison test was used to measure significance. *, adjusted *P < *0.05 and ***, adjusted *P < *0.001. (D) THP-1 cells untreated or pretreated with Wortmannin (1 μM) for 1 h were infected with strain 201 (MOI 40 for 4 h) and stained with MitoSOX Red. Representative confocal images from three independent experiments were shown. Scale bar = 50 μm. The relative % of the MitoSOX Red positive cells were determined (*n* ≈ 200). Data are reported as the mean ± SD from three independent experiments. One-way ANOVA followed by Tukey’s multiple-comparison test was used to measure significance. *, adjusted *P < *0.05 and ***, adjusted *P < *0.001. (E) THP-1 cells/mouse BMDMs untreated or pretreated with Wortmannin (1 μM) for 1 h were infected with strain 201 (MOI 40 for 4 h). Cytosolic mDNA relative to nuclear DNA was analyzed by qPCR. Data are reported as the mean ± SD from three independent experiments. One-way ANOVA followed by Tukey’s multiple-comparison test was used to measure significance. *, adjusted *P < *0.05; **, adjusted *P < *0.01; and ***, adjusted *P < *0.001.

Mitophagy is critical for mitochondrial quality control. Transmission electron microscopy showed that mitochondria were enclosed by a characteristic double-membrane structure, called a phagophore or mitophagosome, in THP-1 cells infected by Y. pestis strain 201 (Fig. 2A). When autophagy is activated, LC3I is converted to LC3 II through modification with phosphatidylethanolamine (PE), and this conversion indicates autophagosome formation. We found that the LC3 II/LC3 I ratio was significantly increased in THP-1 cells infected with strain 201 (Fig. S2A and B), suggesting the autophagy process was activated after Y. pestis infection. When mitophagy is activated, the autophagy marker protein LC3 can be recruited to mitochondria. To confirm that mitochondria were captured by mitophagy post-Y. pestis infection, localization between LC3B and mitochondria was analyzed using immunofluorescent microscopy in THP-1 cells infected with strain 201 or Δ*yscI*. LC3B protein levels increased significantly and a substantial fraction of LC3B were found to be colocalized with COX IV in 201-infected or CCCP-treated THP-1 cells, but not in Δ*yscI*-infected cells (Fig. 2B and C). Similar differences in the colocalization of LC3B with mitochondria between 201- and Δ*yscI*-infected cells were found in mouse macrophage RAW264.7 cells (Fig. S2C and D). These data suggest that mitophagy can be induced in human and mouse macrophages by Y. pestis infection in a T3SS-dependent manner. To identify the role of autophagy in relieving mitochondrial damage caused by Y. pestis infection, cells were treated with Wortmannin, a selective PI3K inhibitor that blocks autophagy formation. Inhibiting autophagy by Wortmannin treatment further increased mROS accumulation and mDNA release in THP-1 cells infected with strain 201 (Fig. 2D and E). In addition, in 201-infected mouse BMDMs, inhibiting autophagic activity with Wortmannin also significantly increased mDNA release (Fig. 2E). These data demonstrate that T3SS is essential for Y. pestis-induced mitophagy, maintaining mitochondrial homeostasis during plague infection.

### YopH plays a crucial role in *Y. pestis*-induced mitochondrial dysfunction and mitophagy.

**FIG 3:**
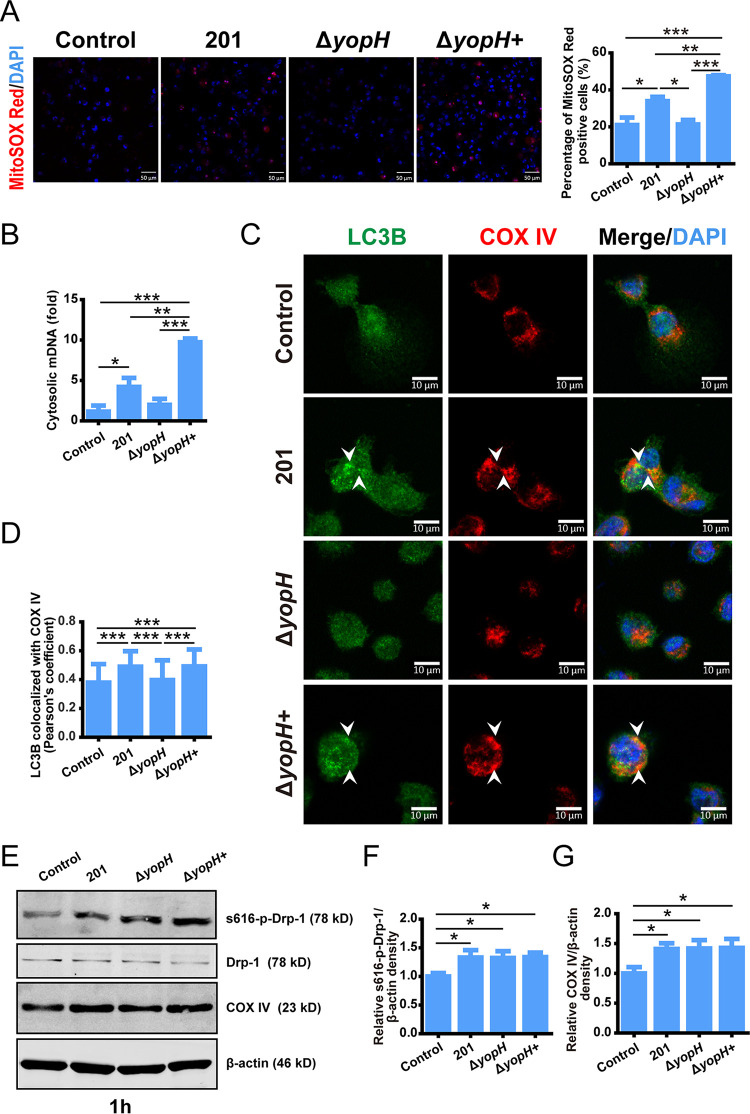
*yopH* deletion decrease mitochondrial damage and mitophagy caused by Y. pestis. (A) THP-1 cells were infected with strain 201, Δ*yopH*, and Δ*yopH+* (MOI 40 for 4 h) and stained with MitoSOX Red. Representative confocal images from two independent experiments were shown. Scale bar = 50 μm. The relative % of the MitoSOX Red positive cells were determined (*n* ≈ 200). Data are reported as the mean ± SD from two independent experiments. One-way ANOVA followed by Tukey’s multiple-comparison test was used to measure significance. *, adjusted *P < *0.05; **, adjusted *P < *0.01; and ***, adjusted *P < *0.001. (B) THP-1 cells were infected with strain 201, Δ*yopH*, and Δ*yopH+* (MOI 40 for 4 h). Cytosolic mDNA relative to nuclear DNA was analyzed by qPCR. Data are reported as the mean ± SD from three independent experiments. One-way ANOVA followed by Tukey’s multiple-comparison test was used to measure significance. *, adjusted *P < *0.05; **, adjusted *P < *0.01; and ***, adjusted *P < *0.001. (C) THP-1 cells were infected with strain 201, Δ*yopH*, and Δ*yopH+* (MOI 40 for 4 h), then stained for LC3B (green) and mitochondrial marker protein COX IV (red). Nuclei were stained with DAPI. Representative confocal images from two independent experiments were shown. Arrowheads indicated the colocalization of LC3B with mitochondria. Scale bar = 10 μm (D) Pearson's coefficient values for LC3B and COX IV colocalization in (C). The average Pearson's coefficients ± SD were calculated for 50 cells from two independent experiments by ImageJ 1.53e software (National Institutes of Health). One-way ANOVA followed by Tukey’s multiple-comparison test was used to measure significance. ***, adjusted *P < *0.001. (E) THP-1 cells were infected with the indicated strains (MOI 40 for 1 h). Immunoblotting analyzed the cell lysates for S616-p-Drp1, Drp-1, or COX IV. The relative density of S616-p-Drp1 (F) and COX IV (G) was determined using Quantity One 4.6.2 software (Bio-Rad, Hercules, CA). *n* = 3, One-way ANOVA followed by Tukey’s multiple-comparison test was used to measure significance. *, adjusted *P < *0.05.

YopH, one of the Yops delivered by T3SS, decreases the ΔΨm, leading to programmed cell death (22). To assess the effect of YopH in Y. pestis-induced mitochondria dysfunction and damage, a *yopH* deletion mutant, Δ*yopH*, was constructed using 201 as the parent strain. The identification results of Δ*yopH* and the complemented strain Δ*yopH+* are shown in Fig. S3 in the supplemental material. THP-1 cells were then infected with Y. pestis strain 201, Δ*yopH*, and Δ*yopH+.* THP-1 cells infected with Δ*yopH* had decreased mROS production and mDNA release compared to cells infected with strain 201 or the complemented strain Δ*yopH+* (Fig. 3A and B). In line with these observations, mitophagy activation was absent in Δ*yopH*-infected cells (Fig. 3C), suggesting that mitochondrial dysfunction in 201-infected cells disappeared due to YopH deletion. Furthermore, there was less colocalization of the LC3B protein with mitochondria in Δ*yopH*-infected THP-1 cells than in 201- or Δ*yopH+*-infected cells (Fig. 3D). Hence, these results indicate that YopH is a vital player in Y. pestis-induced mROS accumulation, mDNA release, and mitophagy activation.

Furthermore, we analyzed Drp-1 phosphorylation and COX IV protein expression post-Y. pestis strain 201, Δ*yopH*, and Δ*yopH+* infection. The levels of S616-p-Drp1 and COX IV increased in the 201- and Δ*yopH-*infected cells in comparison to that in the mock-infected cells at 1 hpi (Fig. 3E to G). However, there were no differences among the strain 201-, Δ*yopH*-, and Δ*yopH*+-infection groups. These data demonstrated that YopH has no effect on Drp-1 phosphorylation or COX IV protein expression during Y. pestis infection.

### *Y. pestis* induces host mitophagy via Pink1/Parkin-independent pathway.

**FIG 4:**
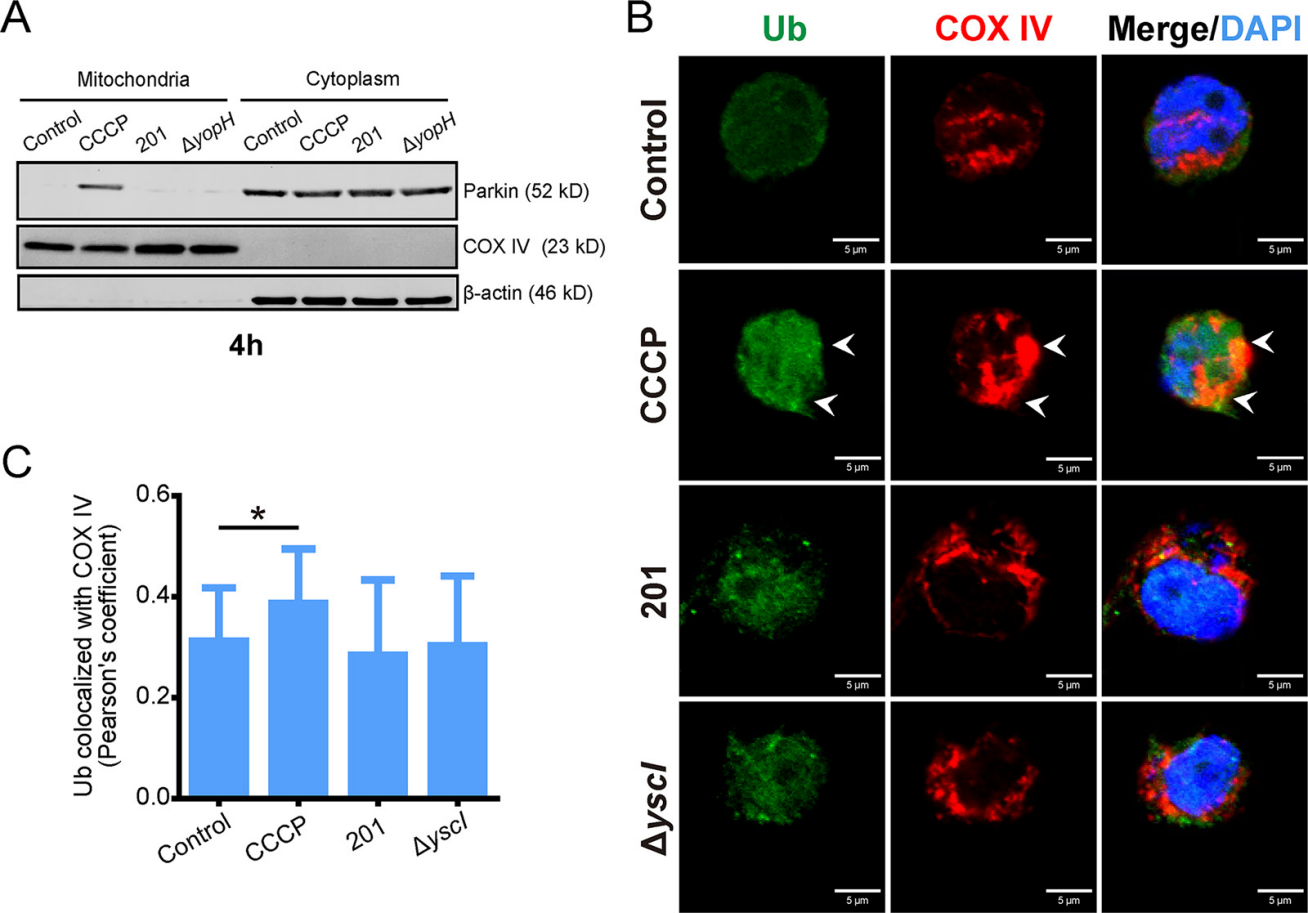
Mitophagy induced by Y. pestis infection is independent of the Pink/Parkin pathway. (A) Parkin subcellular location in THP-1 cells infected with the indicated strains (MOI 40 for 4 h) or CCCP (30 μM) stimulation for 4 h. Mitochondrial and cytoplasmic protein fractions of THP-1 cells were analyzed for Parkin by immunoblotting. Data are representative of three independent experiments. (B) THP-1 cells were infected with the indicated strains (MOI 40 for 4 h), then stained for Ubiquitin (Ub, green) and mitochondrial COX IV (red). Nuclei were stained with DAPI. CCCP (10 μM, 1 h) treatment was used as a positive control. Representative confocal images from two independent experiments are shown. Arrowheads indicate the colocalization of ubiquitin with mitochondria. Scale bar = 5 μm. (C) Pearson’s coefficient values for colocalization of ubiquitin and COX IV in (B). The average Pearson’s coefficients ± SD were calculated for 30 cells from two independent experiments using ImageJ 1.53e software (National Institutes of Health). One-way ANOVA followed by Tukey’s multiple-comparison test was used to measure significance. *, adjusted *P < *0.05.

The Pink1/Parkin pathway is critical for mitophagy induced by CCCP. Cells treated with CCCP exhibited recruitment of Parkin to mitochondria and protein ubiquitination of mitochondria (10). However, no Parkin recruitment (Fig. 4A) or mitochondrial ubiquitination (Fig. 4B and C) were found in cells infected with Y. pestis, suggesting that Y. pestis induces host mitophagy via a Pink1/Parkin-independent pathway.

### mROS restrict *Y. pestis* intracellular survival.

**FIG 5:**
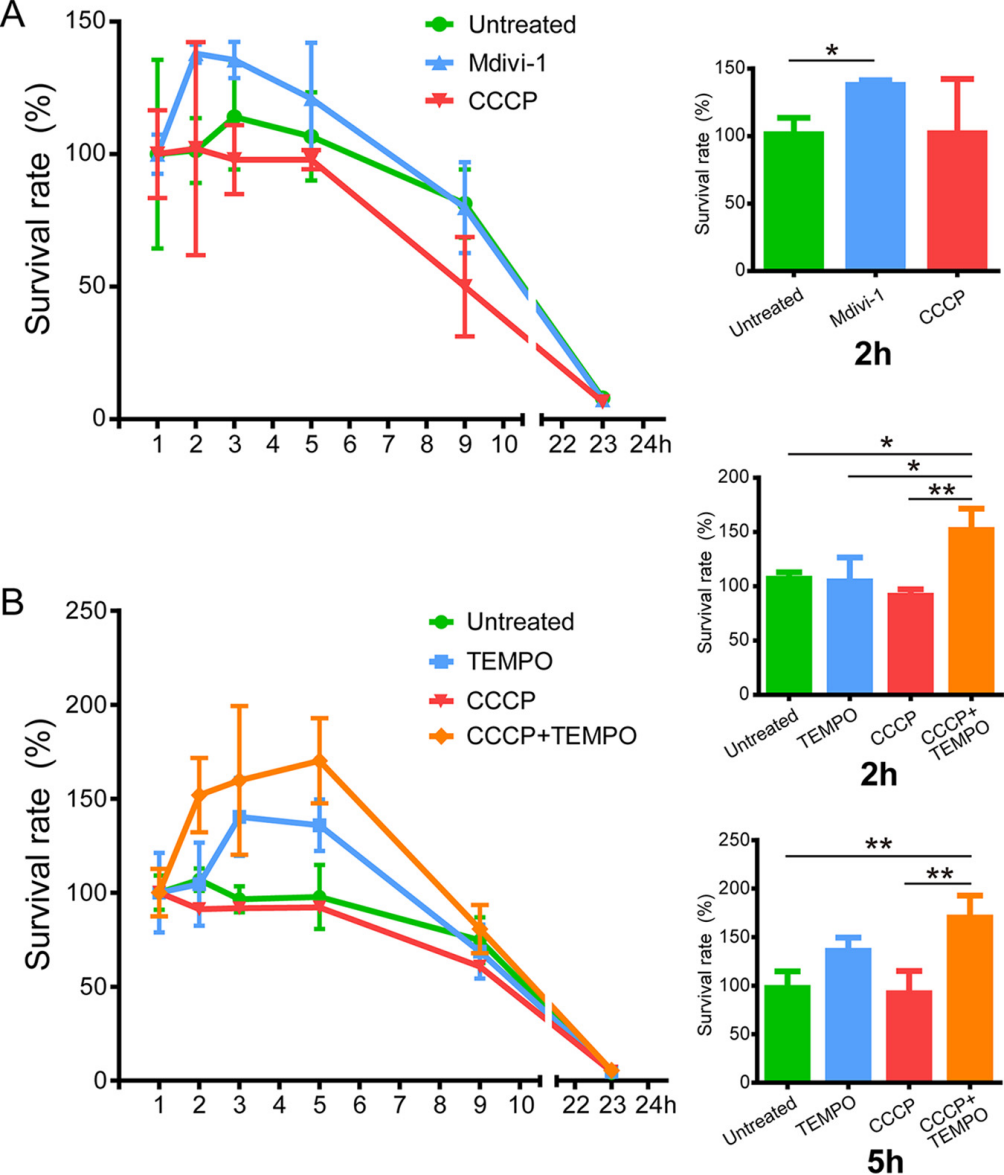
mROS released by damaged mitochondria inhibit the intracellular survival of Y. pestis. (A) Y. pestis strain 201 intracellular survival in THP-1 cells untreated or pretreated with Mdivi-1 (50 μM) or CCCP (10 μM). MOI = 5. Left panel, intracellular strain survival rate from 1 to 23 hpi; right histogram, survival rate at 2 hpi. Data are representative of three to five independent experiments. A two-tailed unpaired Student’s *t* test was used to measure significance. *, adjusted *P < *0.05. (B) Y. pestis strain 201 intracellular survival in THP-1 cells untreated or pretreated with Mito-TEMPO (500 μM) or CCCP (10 μM). MOI = 5.Left panel, survival rate from 1 to 23 hpi; right histogram, survival rates at 2 and 5 hpi. Data are representative of three to five independent experiments. One-way ANOVA followed by Tukey’s multiple-comparison test was used to measure significance. *, adjusted *P < *0.05 and **, adjusted *P < *0.01.

mROS have an essential role in controlling infection of intracellular bacterial pathogens (1, 29). The effects of mROS accumulation on the intracellular survival of Y. pestis were assessed. After treatment with Mdivi-1, CCCP, or Mito-TEMPO, THP-1 cells were infected with strain 201. Intracellular bacterial survival percentages were measured using a gentamicin protection assay followed by plating cell lysates on agar plates. The number of live bacteria in THP-1 cells at 1 hpi was designated the initial value. The survival percentages at 1, 2, 3, 5, 9, and 23 hpi were calculated by dividing the corresponding live bacterial cell numbers to that at 1 hpi. As shown in Fig. 5A, inhibiting mitochondrial fission with Mdivi-1 enhanced the intracellular survival of strain 201 in THP-1 cells at 2 hpi. In contrast, CCCP treatment, which strongly induces mitochondria damage and mROS production, reduced strain 201 survival, but not significantly, during cell infection. The intracellular survival of strain 201 was also improved by scavenging mROS with Mito-TEMPO, but not significantly (Fig. 5B). Moreover, Mito-TEMPO reversed the inhibitive effects of CCCP on intracellular pathogen survival and enhanced survival of 201 in THP-1 cells could even be found at 2 and 5 hpi. Therefore, inhibiting mROS release into the cytoplasm by Mdivi-1 and scavenging mROS by Mito-TEMPO improved the intracellular survival of strain 201 at early time points post-cell infection, whereas mROS aggravation caused by CCCP slightly reduced strain 201 survival during cell infection. These findings suggested that mROS might be involved in the clearance of Y. pestis in macrophages.

### Autophagy activation and mitochondrial fission suppression have significant antagonism to YopH-induced apoptosis.

**FIG 6:**
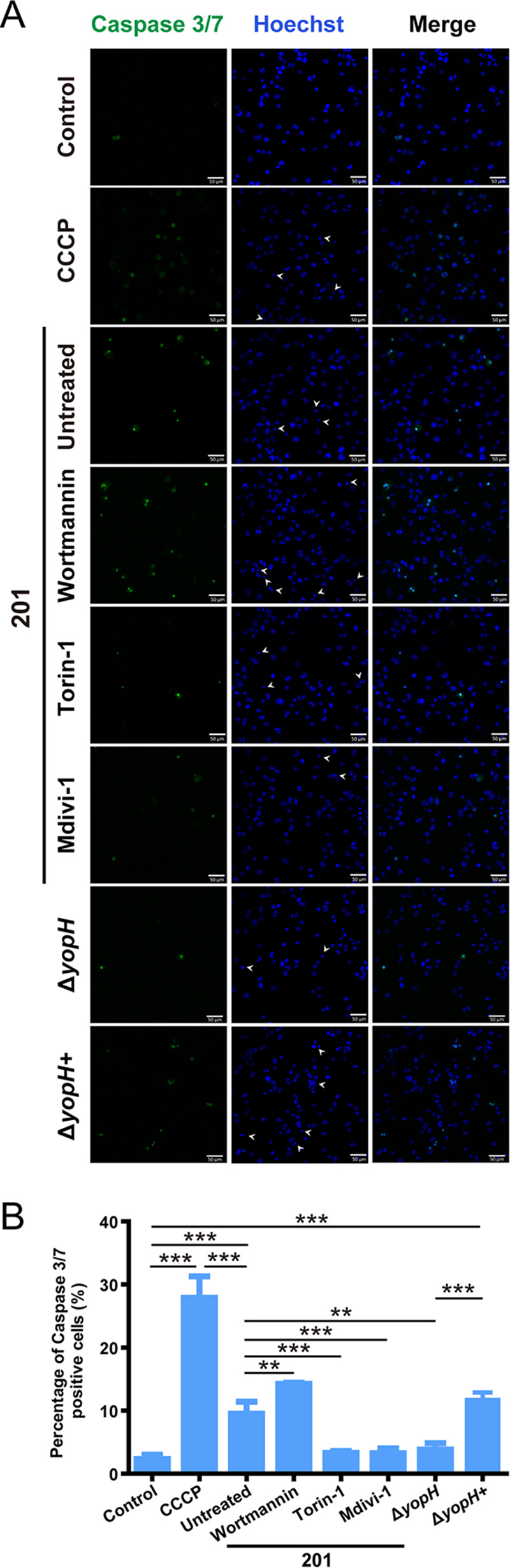
Activating autophagy or inhibiting mitochondrial fission can relieve YopH-induced apoptosis. (A) THP-1 cells, untreated or pretreated with Wortmannin (1 μM), Torin-1 (500 nm), or Mdivi-1 (50 μM) for 1 h, were infected with the indicated strains (MOI = 40 for 4 h) and stained with CellEvent Caspase 3/7 Green ReadyProbes and Hoechst 33342. CCCP (30 μM, 4 h) treatment was used as a positive control. Representative images of three independent experiments are shown. Caspase 3/7-positive cells show green fluorescence signals. Arrowheads indicate condensed or fragmented nuclei of apoptotic cells. Scale bar = 50 μm. (B) The percentage of caspase 3/7-positive cells in (A) was determined (*n* ≈ 200). Data are reported as means ± SD from three independent experiments. One-way ANOVA followed by Tukey’s multiple-comparison test was used to measure significance. **, adjusted *P < *0.01 and ***, adjusted *P* < 0.001.

Combined with previously published results, these data indicate that YopH-induced mitochondrial dysfunction leads to mROS accumulation, the release of mitochondrial contents, and ΔΨm collapse, all of which are proapoptotic stimuli (8, 22). Thus, we next analyzed the effect of autophagy and mitochondrial fission on apoptosis induced by mitochondrial damage during Y. pestis infection. To evaluate cell apoptosis, we analyzed the activity of caspase 3/7 and nuclear morphology. (Fig. 6A and B). Apoptotic cell numbers increased after CCCP treatment or strain 201 and Δ*yopH+* infection compared to that after strain Δ*yopH* infection, confirming that YopH promotes apoptosis of the infected THP-1 cells. In addition, the results showed that inhibiting autophagic activity with Wortmannin increased apoptosis in 201-infected THP-1 cells, while activating autophagy with Torin-1 (mTOR inhibitor) dramatically decreased Y. pestis-induced apoptosis. Moreover, Y. pestis-induced apoptosis was also decreased in THP-1 cells treated by Mdivi-1, which inhibits mitochondrial fission and mitochondrial content release. Treatment with Wortmannin, Torin-1, or Mdivi-1 did not affect caspase 3/7 activity or nuclear morphologic changes (data not shown) in mock-infected cells. Thus, activating autophagy or inhibiting mitochondrial fission are effective measures for suppressing YopH induced mitochondria-dependent apoptosis post-Y. pestis infection.

## DISCUSSION

**FIG 7:**
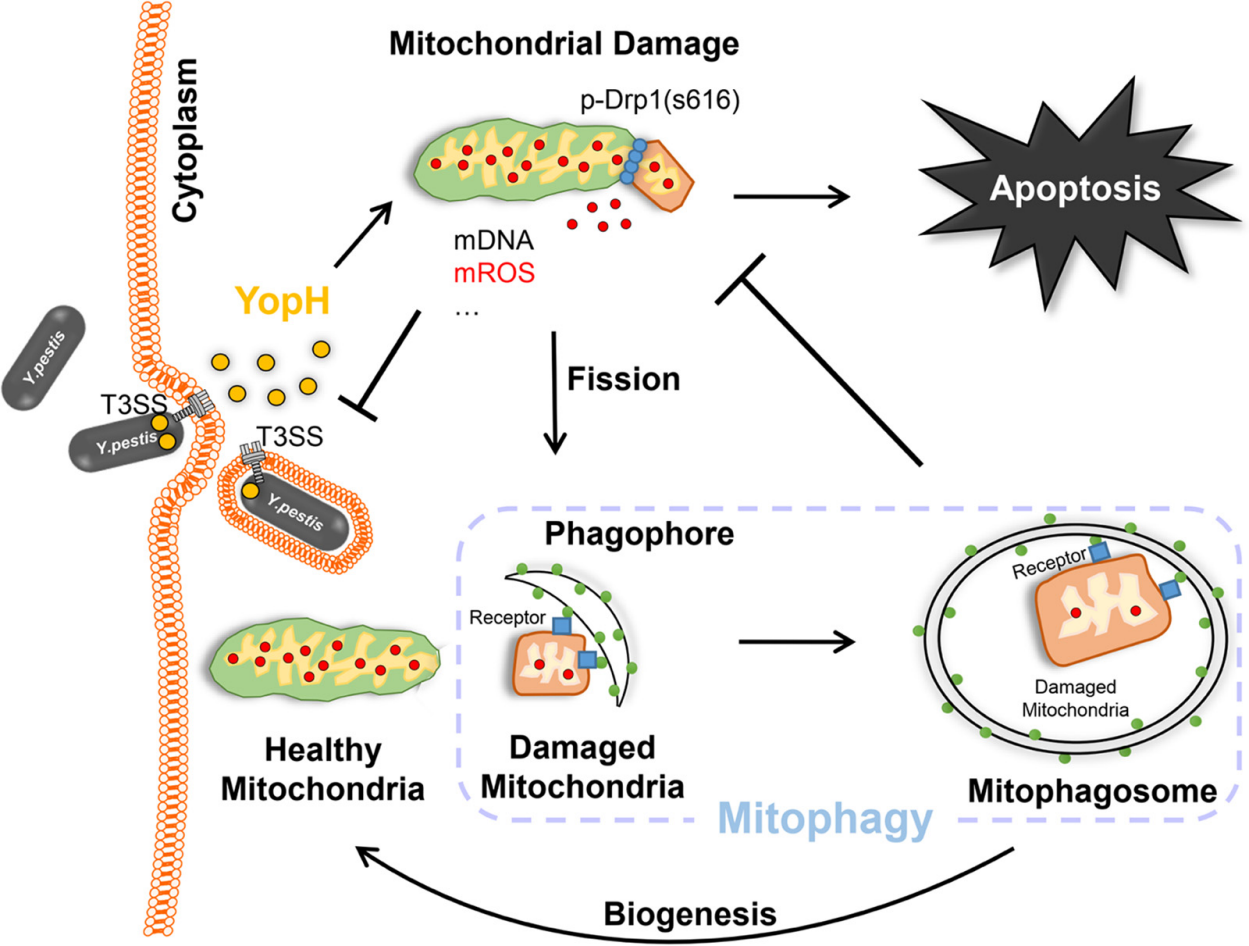
Schematic representation of mitophagy regulation on mitochondrial homeostasis and the bactericidal activity of mROS in Y. pestis*-*infected macrophages. Mitochondrial dysfunction is caused by the YopH effector from Y. pestis, promoting mROS-mediated bactericidal activity and apoptosis of infected macrophages. Subsequently, mitophagy is activated to clear damaged mitochondria, recover mitochondrial homeostasis, and relieve mROS excess.

This study reports that *Y. pestis*-induced mitophagy can eliminate the dysfunctional mitochondria caused by the pathogen’s YopH protein to control the balance of mitochondrial homeostasis and mROS-induced bactericidal activity in macrophages. As shown in Fig. 7, using YopH delivered by T3SS, Y. pestis induces macrophage mitochondrial fragmentation, abnormal mROS accumulation, and mitochondrial content release into the cytoplasm, resulting in the activation of mROS-mediated bactericidal activity to kill intracellular Y. pestis and the promotion of host cell apoptosis. Moreover, host Drp-1 is required for mitochondrial contents to be released to the cytoplasm during Y. pestis infection, suggesting that the mitochondrial dysfunction caused by Y. pestis is not based on the disruption of the mitochondrial structure. On the other hand, *Y. pestis*-induced host mitophagy is responsible for clearing damaged mitochondria and relieving mitochondria-dependent cell death in plague infection. Finally, it may be concluded that Y. pestis-induced mitochondrial damage could be a benefit to the pathogen by inducing damage to the immune cells. Mitophagy is responsible for clearing infection-induced dysfunctional mitochondria to repair immune cells. However, the balance of mitochondrial damage versus the mitophagy that contributes to plague pathogenesis needs further investigation. We speculate that Y. pestis promotes immune cell damage by disrupting mitophagy.

Mitochondrial homeostasis is very sensitive to changes in the cell environment and stress states, thus becoming the target of many pathogenic microorganisms, such as hepatitis C virus, COVID-19, Pseudomonas aeruginosa, and L. monocytogenes (6, 25, 30, 31). This study reports, for the first time, that Y. pestis infection causes severe mitochondrial damage, including mitochondrial fragmentation, abnormal mROS accumulation, and mitochondrial content release into the cytoplasm in macrophages. Y. pestis-induced mitochondrial damage may be a neglected mechanism for this bacterium to influence or damage macrophages. Whether Y. pestis-induced mitochondrial damage contributes to evasion of clearance by professional phagocytes in the lymph nodes during the early stage of infection merits further exploration.

The production of mROS is associated with the bactericidal activity of macrophages, and some pathogens have evolved the capability to evade this host defense mechanism (4). mROS production is triggered by the TLR/TRAF6 pathway in Salmonella infection (or the IFN-γ/STAT1 pathway in *Listeria* infection), contributing to macrophage clearing of the bacteria (1, 32). Nonetheless, infection-induced mROS accumulation seems to be a double-edged sword. Tumor necrosis factor excess induces mROS accumulation in Mycobacterium-infected macrophages through RIP1-RIP3-dependent pathways, initially increasing bactericidal activity but ultimately resulting in cell death and dissemination of the pathogen (33). This study showed that mROS accumulated in macrophages infected with Y. pestis. Both inhibiting mROS release using Mdivi-1 or scavenging mROS using Mito-TEMPO enhanced the intracellular survival of strain 201 at the early time point post-cell infection. Comparatively, enhanced mROS production post-CCCP treatment slightly reduced the survival of strain 201 in macrophages during cell infection. Combined with previously published results, this study proposes that Y. pestis-induced mROS production enhances the antibacterial activity of macrophages against intracellular Y. pestis. Then, mitophagy was activated to eliminate dysfunctional mitochondria and relieve the side effects of mROS accumulation.

Mitophagy is a critical mitochondrial quality control mechanism for maintaining mitochondrial and cellular homeostasis. The Pink1/Parkin pathway plays a crucial role in ubiquitin-dependent mitophagy (10). However, Y. pestis did not induce Parkin or ubiquitin recruitment to mitochondria, indicating that the Pink1/Parkin pathway might not be involved in the mitophagy caused by Y. pestis. Accumulating evidence suggests that some mitophagy receptors (like BNIP3, NIX/BNIP3L, FUNDC1, and BCL2L13) bypass the need for ubiquitin to link the forming mitophagosome with damaged mitochondria (10). Recently, the intracellular bacterial pathogen L. monocytogenes was found to exploit the mitophagy receptor NLRX1 to manipulate mitophagy for survival (6). However, the role of a potential mitophagy receptor in inducing Pink1/Parkin-independent mitophagy during Y. pestis infection remains to be determined.

By altering COX subunit composition, host cells could optimize the efficiency of respiration to adapt to a hypoxic environment (34). Mitochondrial dysfunction, which includes abnormal COX expression, can lead to ROS accumulation (27, 28). We found that COX IV protein expression and mROS production increased in Y. pestis-infected cells. This suggests that Y. pestis affects the central players of the respiratory chain, leading to abnormal mROS accumulation. Similarly, hepatitis C virus core protein directly associates with the mitochondrial outer membrane to reduce complex I activity, leading to the inhibition of mitochondrial electron transport and increasing ROS production (35).

YopH, a protein tyrosine phosphatase, inhibits integrin-mediated bacterial phagocytosis (18, 19) and inactivates the PRAM-1/SKAP-HOM and SLP-76/Vav/PLCg2 signal transduction axes to affect Ca^2+^ flux (36–38). The fine modulation of mitochondrial Ca^2+^ homeostasis plays a fundamental role in many cellular processes involving mitochondria, and compromised mitochondrial Ca^2+^ homeostasis is often associated with mitochondrial dysfunction (39–41). Whether these known activities of YopH contribute to the mitochondrial damage caused by plague infection is worth further investigation, which will help to determine whether the reduced mitochondrial damage caused by the *yopH* mutant is a direct or indirect result of changed phagocytosis or Ca^2+^ signaling caused by YopH.

Mitochondria undergo continuous cycles of fission and fusion to promote regulate quality control and mitigate organelle stress (42). Phosphorylation at serine 616 induces Drp-1 localization to the fission site on the mitochondrion, which then induces mitochondrial fission (26). We found that a functional T3SS was necessary for Y. pestis to promote the phosphorylation at serine 616 of Drp-1. However, YopH deletion has no effect on the Drp-1 phosphorylation induced by Y. pestis infection, although YopH deletion can decrease mitochondrial damage. This suggests that another Yops protein may have a potential effect on the disruption of mitochondrial homeostasis, most likely though regulating the phosphorylation of Drp-1 protein. Similarly, VacA of Helicobacter pylori (43) as well as OmpA of Acinetobacter baumannii (44) induce disruption of mitochondrial morphological dynamics through Drp1-mediated fission. Moreover, Drp-1 inhibitor Mdivi-1, which inhibits mitochondrial fission to block mitochondrial content release, has shown possible therapeutic function in a range of disease (45) and infection models (45, 46). Given that Mdivi-1 treatment can relieve Y. pestis*-*induced cell death, manipulating mitochondrial fission may have a protective effect against Y. pestis-induced cellular dysfunction and tissue injury in clinical medicine and recovery. However, Mdivi-1 treatment reduced Y. pestis-induced cell death but also increased bacterial burdens (Fig. 5A). Therefore, manipulating mitochondrial fission in this system could worsen disease through greater Y. pestis replication. The balance of effectively relieving mitochondrial dysfunction and minimizing the negative impacts of Y. pestis burdens in Mdivi-1 treatment need to be addressed.

Our data deepen the understanding of the effects of Y. pestis pathogenesis on mitochondrial dysfunction, inducing apoptosis and damage to immune cells during infection. These findings reveal the regulation effect of mitophagy in the macrophage on the balance between mitochondrial homeostasis and mROS-mediated bactericidal activity in response to Y. pestis infection. The mitophagy activation mechanism involved in Y. pestis-induced Pink1/Parkin-independent mitophagy remains to be determined in future studies.

## MATERIALS AND METHODS

### Bacterial strains, cells, and growth conditions.

Y. pestis strain 201 belongs to the biovar Microtus, which is highly virulent to mice but avirulent to humans (23). The Y. pestis strain 141 is a biovar Antiqua strain which is highly virulent in mice and humans (24). The *yscI* mutant strain Δ*yscI*, derived from strain 201, was constructed in our previous studies (47). The *yopH* mutant Δ*yopH* and the complemented strain Δ*yopH+* were constructed in this study. The glycerol-preserved strains were inoculated in 5 mL Luria-Bertani (LB) broth and allowed to grow at 26°C (for cell infection) or 37°C (for Western blotting detection of YopH protein in the *yopH* mutant and the complemented strain) with shaking at 200 rpm for 16 h. The bacteria and plasmids used in this study are listed in Table 1.

**TABLE 1:** Bacterial strains and plasmids used in this study^*a*^

Strain/plasmid	Characteristics	Source
Strain		
201	Wild-type Y. pestis biovar Microtus strain	23
Δ*yscI*	Strain 201 with *yscI* replaced by Kan cassette, Kan^r^	47
Δ*yopH*	Strain 201 with *yopH* replaced by Kan cassette, Kan^r^	This study
Δ*yopH+*	201 Δ*yopH* strain carrying plasmid pACYC184-*yopH* (Cm^r^)	This study
141	Wild-type Y. pestis biovar Antiqua strain	24
		
Plasmid		
pKD4	Kan^r^	48
pKD46	Temp-sensitive plasmid expressing λ-Red recombinase under the control of arabinose, Amp^r^	48
pACYC184-*yopH*	pACYC184 carrying the entire *yopH* gene, Cm^r^	This study

a Kan, kanamycin; Amp, ampicillin; Cm, chloramphenicol.

THP-1 cells were maintained in RPMI 1640 medium containing 10% fetal bovine serum (FBS), streptomycin-penicillin, and l-glutamine. RAW264.7 cells and mouse BMDMs were maintained in Dulbecco’s modified Eagle’s medium (DMEM) supplemented with 10% FBS, streptomycin-penicillin, and l-glutamine at 37°C in a 5% CO_2_ incubator.

### Cell infection.

THP-1 cells were primed with phorbol myristate acetate (PMA, 100 ng/mL, p8139; Sigma-Aldrich, Louis, MO) for 48 h before infection. THP-1 cells, RAW264.7 cells, and mouse BMDMs were seeded onto 12-well plates at 5 × 10^5^/mL concentration. Y. pestis strains were grown in LB broth at 26°C until reaching an optical density at 620 nm (OD_620_) of 1.0, and bacterial cells were collected by centrifugation and resuspended in the indicated cell culture medium. The cells were then infected with Y. pestis strains at a multiplicity of infection (MOI) of 5 (for analyzing the intracellular survival capabilities of Y. pestis) or 40 (for other experiments). The infected cells were centrifuged briefly to promote the adhesion of bacteria to the cells before incubation at 37°C in a 5% CO_2_ incubator. The cell samples were used for subsequent analysis at the indicated time points postinfection.

### Cell treatment with small molecule compound.

The Mito-TEMPO (500 μM, SML0737, Sigma-Aldrich, Louis, MO), Wortmannin (1 μM, tlrl-wtm, Invivogen, San Diego, CA), Mdivi-1 (50 mM, M0199, Sigma-Aldrich), CCCP (10 μM or 30 μM, C2759, Sigma-Aldrich) and Torin-1 (500 nm, inh-tor1, Invivogen) was added directly to the cell culture medium for indicated times before infection.

### Construction of the YopH mutant strain.

The strain Δ*yopH* was constructed by a λ-Red recombination system (48, 49). Briefly, the kanamycin (Kan) resistance cassette was amplified from the pKD4 plasmid using the primers YopH-kan forward/reverse to include 40-bp homology extensions from the 5′ and 3′ ends of the *yopH* gene. The PCR products were purified and introduced into Y. pestis strain 201 containing pKD46 plasmid by electroporation. Recombinant 201-Kan bacteria were screened and selected on Kan resistance LB agar plates. Allelic replacement of *yopH* by the Kan resistance cassette was verified by PCR (primers: YopH-inner forward/reverse) and Western blotting (YopH antibody), then pKD46 plasmid was eliminated by incubation at 42°C for 16 h. To construct the complemented strain Δ*yopH+*, the full-length *yopH* gene was cloned from Y. pestis strain 201 using the primers YopH-com forward/reverse and ligated into plasmid pACYC184. The recombined plasmid pACYC184-*yopH* was introduced into the strain Δ*yopH*, and the transformants were screened and selected on Chloramphenicol (Cm) resistance LB agar plates. The YopH mutant and complemented strain were confirmed by PCR (primers: YopH-inner forward/reverse) and Western blotting (YopH antibody). The primers used are listed in Table S1 in the supplemental material.

### Immunoblotting and antibodies.

After infection (MOI = 40) or treatment for the indicated time, the cultured cells were rinsed twice in PBS and were lysed in protein loading buffer (50 mM Tris [pH 6.8], 100 mM dithiothreitol, 2% SDS, 0.1% bromophenol blue, and 10% glycerol), and the remaining cells were scraped off the dish and sonicated to shear the DNA and reduce the sample viscosity. Equal amounts of protein were separated by SDS-polyacrylamide gel electrophoresis and transferred to polyvinylidene difluoride membranes. The nonspecific sites were blocked with 5% bovine serum albumin (BSA, B2064, Sigma-Aldrich, Louis, MO) in TBST (50 mM Tris, 150 mM NaCl, and 0.05% Tween 20 adjusted to pH 7.6 using HCl), and the membranes were then incubated with dilutions of the primary antibodies as recommended by the manufacturers. The primary antibodies included the following: anti-LC3B (1:1,000, 3868), anti-DRP1 (1:1,000, 8570) and anti-phospho-DRP1 (Ser616, 1:1,000, 3455) (Cell Signaling Technology, Danvers, MA); anti-COX IV (1:1,000, ab33985), and anti-Parkin (1:1,000, ab77924) (Abcam, Boston, MA); and anti-β-actin (1:2,000, sc-8432, Santa Cruz Biotechnology, Dallas, TX) and anti-YopH (1:2,000, Lab homemade rabbit polyclonal antibody against YopH recombinant protein). The membranes were washed and incubated with IRDye 800CW-conjugated goat anti-rabbit secondary antibodies (1:5,000, 926-32211) or goat anti-mouse secondary antibodies (1:5,000, 926-32210) (LI-COR Biosciences, Lincoln, NE). Images of the immunoblotting results were taken by an Odyssey SA imaging system (LI-COR Biosciences).

### Immunofluorescence.

Cells were cultured on a glass-bottomed cell culture dish (801002, NEST, Wuxi, Jiangsu, China) in the indicated medium and infected with Y. pestis as described above (MOI = 40 for 4 h) or treated with CCCP at the indicated time points. Cells were then washed with PBS and fixed for 15 min at room temperature with 4% paraformaldehyde in PBS, followed by three washes with PBS. After permeabilization with 0.2% Triton X-100 in PBS for 10 min and blocking with 5% BSA in PBS for 1 h, cells were incubated with primary antibodies, including anti-LC3B (1:200, 3868, Cell Signaling Technology, Danvers, MA), anti-COX IV (1:500, ab33985) and anti-Ubiquitin (1:500, ab223378) (Abcam, Boston, MA), and anti-COX IV (1:500, 4850, Cell Signaling Technology) overnight at 4°C. Cells were then washed three times with PBS and incubated for 1 h at room temperature with fluorescent secondary antibody (Cell Signaling Technology), followed by three washes with PBS, incubation with 4′,6-diamidino-2-phenylindole (DAPI) for 5 min, and observation with the Olympus FluoView FV1000 confocal microscope or the Zeiss LSM 880 confocal microscope.

### MitoSOX staining.

MitoSOX staining was performed following the manufacturer’s protocol (Invivogen). Cells were cultured on a glass-bottomed cell culture dish in the indicated medium and infected with Y. pestis as described above (MOI = 40 for 4 h) or treated with CCCP at the indicated times. Then, the cells were incubated with 5 μM MitoSOX Red mitochondrial superoxide indicator (M36008, Invivogen, San Diego, CA) in cell culture medium at 37°C for 10 min, protected from light. After washing with PBS, the cells were incubated with DAPI for 5 min and observed with the Olympus FluoView FV1000 confocal microscope or the Zeiss LSM 880 confocal microscope.

### Electron microscopy.

As described above, cells were grown and infected (MOI = 40 for 1 and 4 h). For electron microscopy, cells were prefixed with 2.5% glutaraldehyde at 4°C overnight, then washed three times with PBS. After postfixation with 1% osmic acid at 4°C for 1.5 h, samples were dehydrated stepwise in a graded series of ethanol and embedded in acrylic resin at 60°C for 48 h. Ultra-thin sections were mounted on nickel grids. Samples were stained with uranyl acetate for 15 min and lead citrate for 10 min, and then rinsed with distilled water. Samples were examined using a 120 kV Hitachi HT7700 transmission electron microscope equipped with a digital camera.

### Measurement of cytoplasmic mDNA release.

mDNA release was measured based on the method previously described by Bronner et al. (50). Briefly, 1 × 10^6^ cells, infected (MOI 40 for 4 h) or treated as indicated, were lysed with 1% NP-40 (IGEPAL CA-630, I8896, Sigma-Aldrich, Louis, MO) on ice for 15 min, then centrifuged at 16,000 × *g* for 15 min at 4°C. Then, a DNA Mini Kit (51306, Qiagen, Hilden, Germany) was used to purify mDNA from the cytosolic fraction according to the manufacturer’s instructions. Quantitative PCR was used to measure cytoplasmic mDNA using a SYBR Green I Master (04887352001, Roche, Indianapolis, IN). The copy number of cytoplasmic mDNA was normalized to that of nuclear DNA as the ratio of DNA encoding MT-CO1 (mitochondrially encoded cytochrome c oxidase I) to nuclear DNA encoding 18S rRNA genes. The primers used are listed in Table S1 in the supplemental material. The copy number of DNA encoding MT-CO1 was measured by quantitative real-time PCR with the same volume of the DNA solution.

### Isolation of mitochondria.

According to the manufacturer’s instructions, mitochondria were isolated from 4 × 10^7^ THP-1 cells using a mitochondria isolation kit (MITOISO2, Sigma-Aldrich, Louis, MO). The mitochondrial protein and cytoplasmic supernatant were harvested for immunoblotting analysis at the indicated time points post-infection or CCCP treatment.

### Survival capabilities of *Y. pestis* in THP-1 cells.

After being primed with PMA (100 ng/mL) for 48 h, THP-1 cells were seeded onto 24-well plates at a 2 × 10^5^/mL concentration. Y. pestis strains were grown in LB broth at 26°C until reaching an OD_620_ of 1.0, and bacterial cells were collected by centrifugation and resuspended in RPMI 1640 medium. The cells were then infected with Y. pestis strains at an MOI of 5, and gentamicin at 30 μg/mL was added to kill extracellular bacteria after 0.5 h of infection. At 1, 2, 3, 5, 9, and 23 hpi, the culture medium from each well was decanted, the cells were thoroughly washed in PBS, and the infected cells were lysed by the addition of sterile distilled water containing 0.1% Triton X-100 for 15 min at room temperature to release the engulfed bacteria. The living bacteria were counted by plating the diluted cell lysates onto the agar plates in triplicate.

### Determination of caspase 3/7 activity.

According to the manufacturer’s protocol, we checked caspase 3/7 protease activity using the CellEvent Caspase 3/7 Green ReadyProbes reagent (R37111, Invivogen, San Diego, CA). After being primed with PMA (100 ng/mL) for 48 h, THP-1 cells were seeded onto a glass-bottomed cell culture dish. After infection (MOI = 40 for 4 h) or treatment as described above, THP-1 cells were stained with fluorescent caspase substrate for 30 min. Then, the cells were incubated with Hoechst 33342 for 5 min and observed with a fluorescence microscope.

### Analysis of nuclear morphology and apoptosis.

After stimulation with PMA (100 ng/mL) for 48 h, THP-1 cells were seeded onto a glass-bottomed cell culture dish. After infection (MOI = 40 for 4 h) or treatment as described above, THP-1 cells were stained with Hoechst 33342 for 5 min and observed with a fluorescence microscope. Apoptotic cells were identified based on characteristic changes, including nuclear condensation, fragmentation, and apoptotic bodies (51).

### Statistical analysis.

Results are expressed as means ± standard deviation. For graphs, all data were analyzed using Microsoft Excel (v.15.0.5415.1000, Microsoft, Redmond, WA) or GraphPad Prism (v.5.0.0, GraphPad Software, La Jolla, CA). The differences between the two samples were analyzed using a two-tailed unpaired Student’s *t* test; differences among three or more groups were analyzed using a one-way analysis of variance (ANOVA) followed by a Tukey’s multiple-comparison test. *P* values of ≤0.05 were considered statistically significant.
