# Supplementary material for: Yersinia pestis-Induced Mitophagy That Balances Mitochondrial Homeostasis and mROS-Mediated Bactericidal Activity
Source: Microbiol Spectr. 2022 Jun 6;10(3):e00718-22. doi: 10.1128/spectrum.00718-22 (PMC9241946; doi:10.1128/spectrum.00718-22)

Supplementary Table 1. Primers used in this study

**Primers**	**Sequence (5’-3’)**
YopH-inner forward	GCACCAAGCACTGTTTCTC
YopH-inner reverse	GTGTTACCGACCTGGATGT
YopH-kan forward	TGTTTATAGTATAGGCGTGTATTTAATTAAGGAGGGAAGCTGTGTAGGCTGGAGCTGCTTC
YopH-kan reverse	TCTTAGTAATTTTATTTACTCATAGGAATAAATATTTACACATATGAATATCCTCCTTAG
YopH-com forward	AAGCTTGAAAACCGCTTCAGCTAATTAC
YopH-com reverse	GGATCCTTAGCTATTTAATAATGGTCGCC
MT-CO1 forward (mouse)	GCCCCAGATATAGCATTCCC
MT-CO1 reverse (mouse)	GTTCATCCTGTTCCTGCTCC
MT-CO1 forward (human)	TTCACCTCCGCTACCATAA
MT-CO1 reverse (human)	GCACTGCAGCAGATCATTT
18S rDNA forward	TAGAGGGACAAGTGGCGTTC
18S rDNA reverse	CGCTGAGCCAGTCAGTGT

SUPPLEMENTARY FIGURE LEGENDS

Supplementary Figure 1. Mitochondrial damage caused by *Y. pestis* infection. (A) Electron micrographs of mitochondrial morphology in U937 cells infected with strain 201 or fully virulent strain 141 for 4 h. The line indicates mitochondrial length. Scale bar = 10 μm. **(B)** The mouse BMDMs untreated or pretreated with Mito-TEMPO (500 μM) for 1 h were infected with strain 201 and Δ*yscI* (MOI 40 for 4 h) and stained with MitoSOX Red. CCCP (30 μM, 4 h) treatment was used as a positive control. Representative confocal images from two to three independent experiments were shown. Scale bar = 50 μm. The relative % of the MitoSOX Red positive cells were determined (n ≈ 150). Data are reported as the mean ± SD from two to three independent experiments. One-way ANOVA followed by Tukey's multiple comparison test was used to measure significance. *adjusted $P < 0.05$, **adjusted $P < 0.01$ and ***adjusted $P < 0.001$.

Supplementary Figure 2. Autophagy is activated in macrophages infected with *Y. pestis*. (A) The immunoblotting of LC3B was used to track the conversion of LC3B I into LC3B II for autophagic activity in the THP-1 cells infected with the strain 201 (MOI 40 for 3 h before harvested). **(B)** The relative density of LC3B II/LC3B I was determined using Quantity One 4.6.2 software (Bio-Rad, Hercules, CA, USA). n=3, a Two-sided unpaired Student's *t*-test was

used to measure significance. *adjusted $P < 0.05$. **(C)** RAW264.7 cells were infected with strain 201 and Δ*yscI* (MOI 40 for 4 h), then stained for LC3B (green) and mitochondrial marker protein COX IV (red). Nuclei were stained with DAPI. Representative confocal images from two independent experiments were shown. Arrowheads indicated the colocalization of LC3B with mitochondria. Scale bar = 5 μm. **(D)** Pearson's coefficient values for colocalization of LC3B and COX IV in (C). The average Pearson's coefficients ± SD were calculated from ~20 cells by ImageJ 1.53e software (National Institutes of Health, USA). One-way ANOVA followed by Tukey's multiple comparison test was used to measure significance. **adjusted $P < 0.01$.

Supplementary Figure 3. Identification of YopH mutant strain. (A) PCR verification of the 201 YopH mutant. The parent strain 201 and complemented strain Δ*yopH*+ genome template PCR product amplified by the *yopH* gene inner primers (YopH-inner forward/reverse) is 218 bp compared to that YopH mutant strain Δ*yopH* is no amplified product. **(B)** YopH protein expression level in the parent strain 201, the YopH mutant strain Δ*yopH* and the complemented strain Δ*yopH*+ that incubated at 37 °C overnight. Total bacterial lysates were immunoblotted with antibodies against YopH protein. At the protein level, the YopH protein was absent in mutant Δ*yopH* and over-expressed in the complemented strain Δ*yopH*+.

SFig. 1

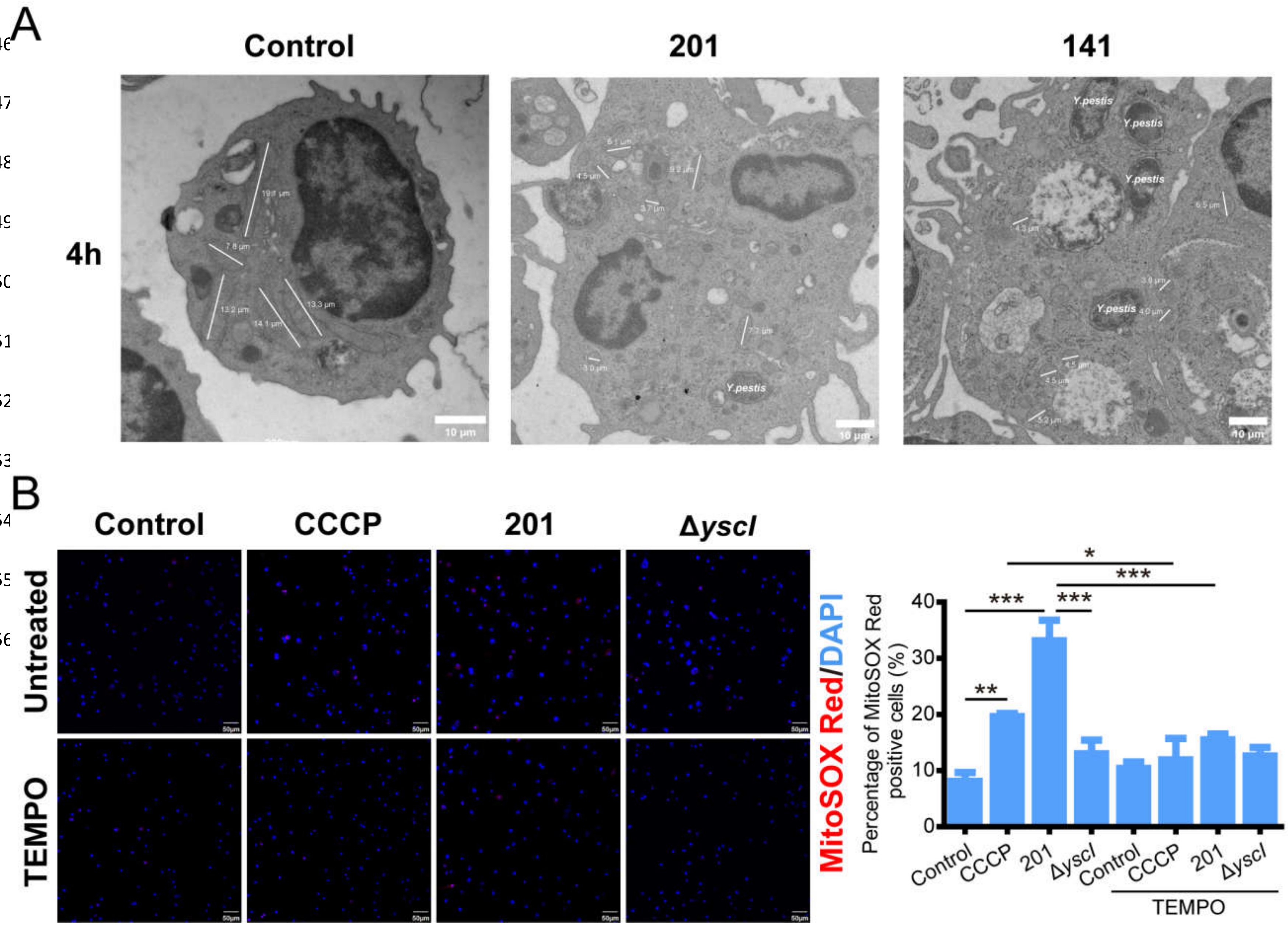
A
Control
201
141
4h
Y.pestis
10 μm
B
Control
CCCP
201
Δyscl
Untreated
TEMPO
50μm
MitoSOX Red/DAPI
Percentage of MitoSOX Red positive cells (%)
0
10
20
30
40
*
**
***
Control
CCCP
201
Δyscl
Control
CCCP
201
Δyscl
TEMPO

SFig. 2

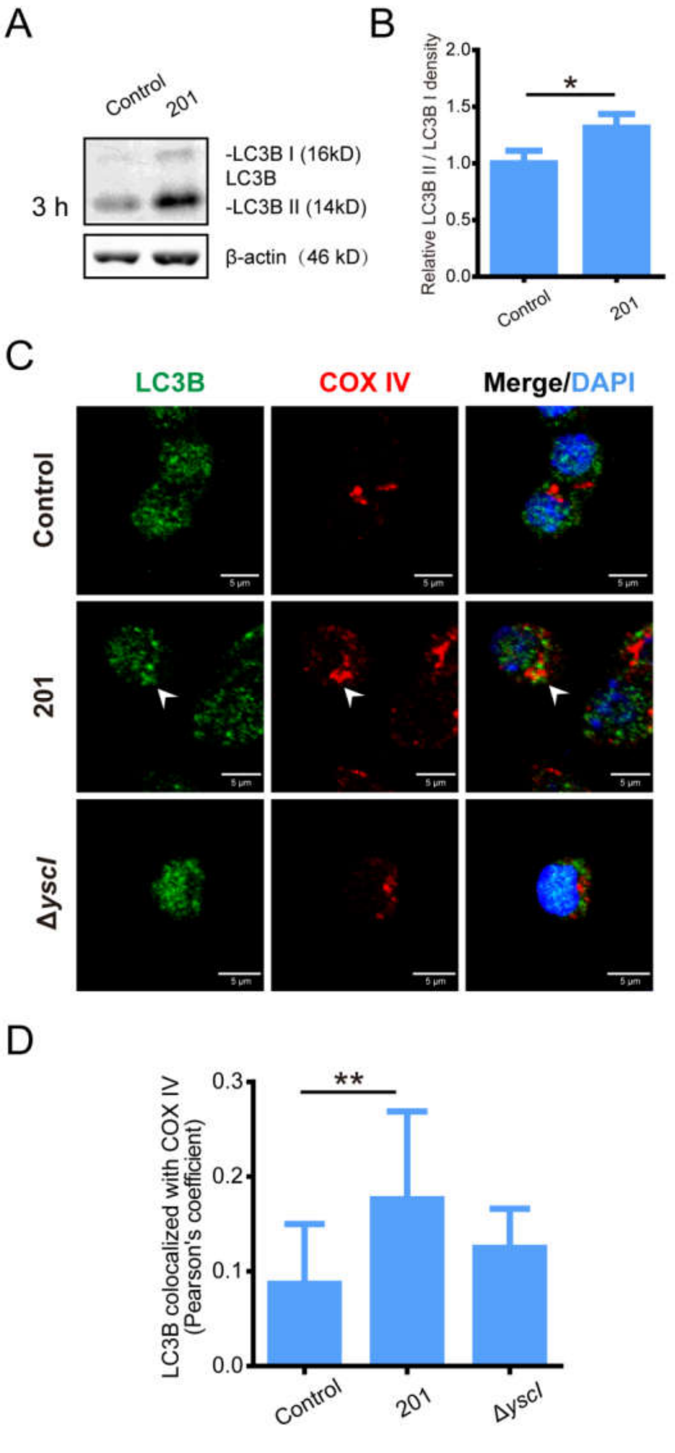

A
Control
201
3 h
-LC3B I (16kD)
LC3B
-LC3B II (14kD)
β-actin (46 kD)
B
Relative LC3B II / LC3B I density
2.0
1.5
1.0
0.5
0.0
*
Control
201
C
LC3B
COX IV
Merge/DAPI
Control
201
Δyscl
5 μm
D
LC3B colocalized with COX IV (Pearson's coefficient)
0.3
0.2
0.1
0.0
**
Control
201
Δyscl

SFig. 3

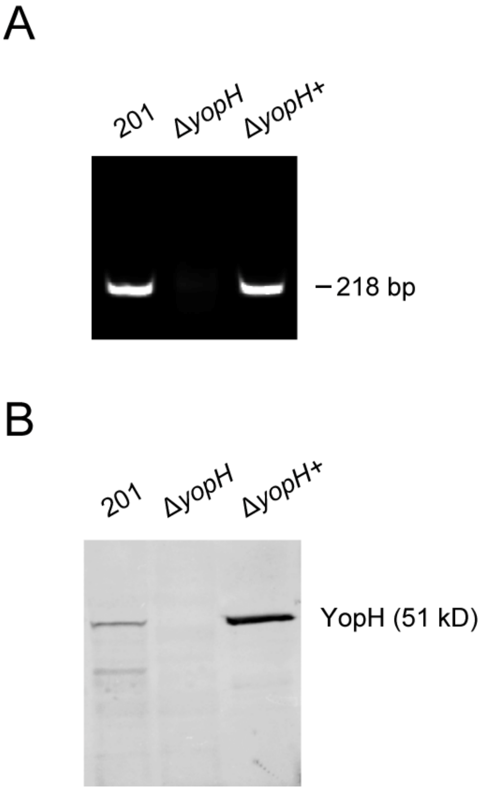

Supplement: Supplemental file 1 — Supplemental material. Download spectrum.00718-22-s0001.pdf, PDF file, 0.3 MB [file spectrum.00718-22-s0001.pdf]
